# Supplementary material for: Causal Relationship between Aging and Anorexia Nervosa: A White-Matter-Microstructure-Mediated Mendelian Randomization Analysis
Source: Biomedicines. 2024 Aug 16;12(8):1874. doi: 10.3390/biomedicines12081874 (PMC11351342; doi:10.3390/biomedicines12081874)
Supplement: Supplementary file 1 [file biomedicines-12-01874-s001.zip › Supplementary Figure/Figure S4/Singe SNP plot of MR analysis for TL to ubm-b-1563.pdf]

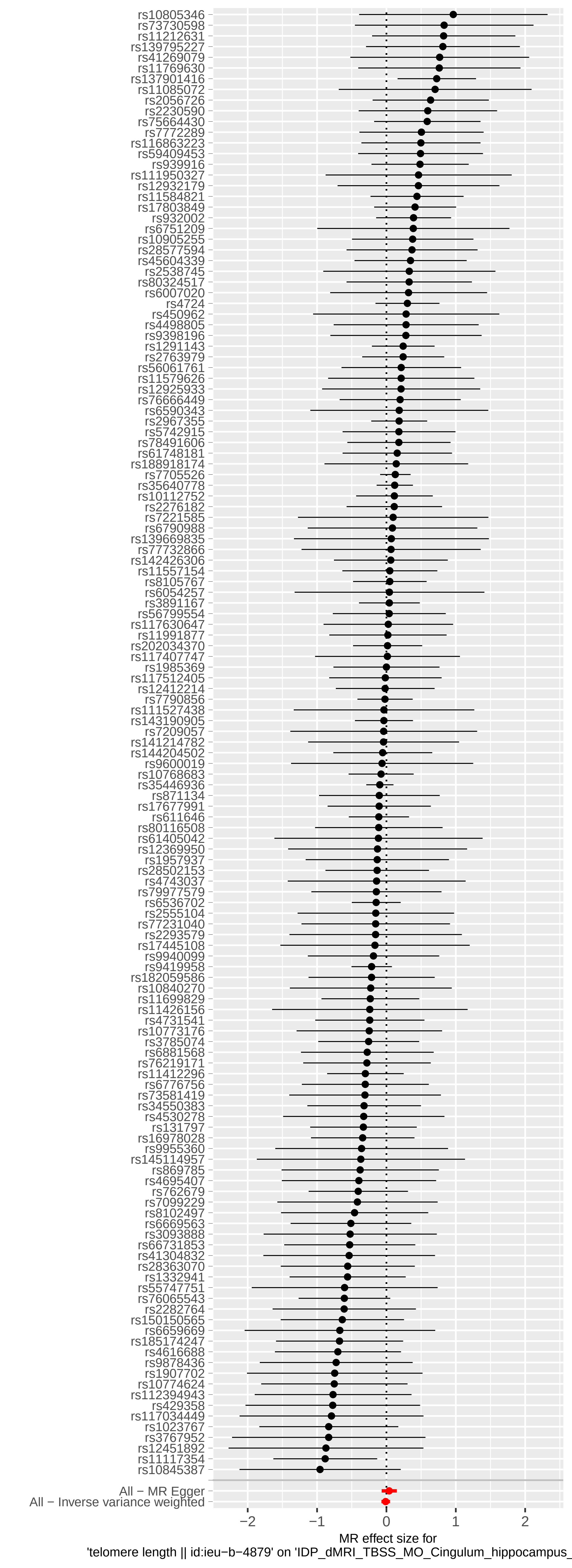
rs10805346
rs73730598
rs11212631
rs139795227
rs41269079
rs11769630
rs137901416
rs11085072
rs2056726
rs2230590
rs75664430
rs7772289
rs116863223
rs59409453
rs939916
rs111950327
rs12932179
rs11584821
rs17803849
rs932002
rs6751209
rs10905255
rs28577594
rs45604339
rs2538745
rs80324517
rs6007020
rs4724
rs450962
rs4498805
rs9398196
rs1291143
rs2763979
rs56061761
rs11579626
rs12925933
rs76666449
rs6590343
rs2967355
rs5742915
rs78491606
rs61748181
rs188918174
rs7705526
rs35640778
rs10112752
rs2276182
rs7221585
rs6790988
rs139669835
rs77732866
rs142426306
rs11557154
rs8105767
rs6054257
rs3891167
rs56799554
rs117630647
rs11991877
rs202034370
rs117407747
rs1985369
rs117512405
rs12412214
rs7790856
rs111527438
rs143190905
rs7209057
rs141214782
rs144204502
rs9600019
rs10768683
rs35446936
rs871134
rs17677991
rs611646
rs80116508
rs61405042
rs12369950
rs1957937
rs28502153
rs4743037
rs79977579
rs6536702
rs2555104
rs77231040
rs2293579
rs17445108
rs9940099
rs9419958
rs182059586
rs10840270
rs11699829
rs11426156
rs4731541
rs10773176
rs3785074
rs6881568
rs76219171
rs11412296
rs6776756
rs73581419
rs34550383
rs4530278
rs131797
rs16978028
rs9955360
rs145114957
rs869785
rs4695407
rs762679
rs7099229
rs8102497
rs6669563
rs3093888
rs66731853
rs41304832
rs28363070
rs1332941
rs55747751
rs76065543
rs2282764
rs150150565
rs6659669
rs185174247
rs4616688
rs9878436
rs1907702
rs10774624
rs112394943
rs429358
rs117034449
rs1023767
rs3767952
rs12451892
rs11117354
rs10845387
All − MR Egger
All − Inverse variance weighted
−2
−1
0
1
2
MR effect size for
'telomere length || id:ieu−b−4879' on 'IDP_dMRI_TBSS_MO_Cingulum_hippocampus_[illegible]
